# Nicotine and heavy metal exposure in recent exclusive cigar users: Insights from the U.S. population assessment of tobacco and health study wave 7 (2022−2023)

**DOI:** 10.1016/j.pmedr.2026.103648

**Published:** 2026-10-03

**Authors:** Mary Rezk-Hanna, Amanda Adolfo, Umme S. Warda, Mary-Lynn Brecht, Neal L. Benowitz

**Affiliations:** a Joe C. Wen School of Nursing, University of California, Los Angeles, Los Angeles, California, United States of America; b School of Medicine, Virginia Commonwealth University, Richmond, Virginia, United States of America; c Pharmacology Research Program, Division of Cardiology, Department of Medicine, University of California, San Francisco, San Francisco, California, United States of America

**Keywords:** Cigar smoking, Biomarkers of exposure, Heavy metals, TNE-2, PATH study

## Abstract

**Objective:**

Population-level biomarker data among recent exclusive cigar users remain limited. We examined nicotine exposure (urinary TNE-2) and heavy metal biomarkers among recent exclusive cigar users, cigarette users and never tobacco users using PATH Wave 7 (2022–2023) data.

**Methods:**

We compared geometric mean urinary cadmium, lead, uranium, cotinine, and TNE-2 among exclusive cigar users (*n* = 79), exclusive cigarette users (*n* = 1192), and never tobacco users (*n* = 691). Users were classified by last use as Today (same-day biospecimen collection), or Yesterday/Day Before (1–2 days earlier). Survey-weighted regression assessed unadjusted and covariate-adjusted group differences.

**Results:**

Cigar users were predominantly male, older (≥55 years), non-Hispanic Black, and had higher prevalence of cardiovascular conditions/risk factors than cigarette users (*p* < 0.05). Compared with never users, same-day cigar users had higher cadmium, uranium, cotinine and TNE-2 (all *p* < 0.01). Cadmium and lead levels were comparable between same-day cigar and cigarette users (p = ns), whereas uranium was higher among cigar users (*p* < 0.05). Adjusted models showed higher cadmium, uranium, and nicotine biomarkers in same-day cigar users, and lead and nicotine biomarkers in yesterday/day-before cigar users, versus never users.

**Conclusions:**

Despite lower nicotine biomarkers, same-day cigar users had cadmium and lead concentrations similar to same-day cigarette smoking, while uranium was higher unadjusted. These findings support cigar regulation and cessation efforts.

## 1 Introduction

Cigars (cigarillos, filtered cigars, and traditional large cigars) are the second most commonly used combustible tobacco product among U.S. adults (Tasdighi et al., 2025). Because they are smoked, cigars deliver numerous toxic and carcinogenic compounds, including tobacco-specific nitrosamines and volatile organic compounds (VOC) linked to cardiopulmonary disease and cancer (Vargees et al., 2023; Pickworth et al., 2018). Population-level data show that use patterns vary by cigar type, with premium cigars used less frequently and by older, non-Hispanic White men, whereas flavored cigars are more prevalent among younger and racially diverse populations, with flavors increasing appeal and facilitating inhalation (Jeon et al., 2023; Corey et al., 2018).

Few studies have examined biomarkers among exclusibe cigar users. Nationally representative analyses from the Population Assessment of Tobacco and Health (PATH) Study Wave 1 (2013–2014) showed that exclusive cigar users had higher nicotine and toxicant biomarker concentrations, including NNAL (tobacco-specific nitrosamine), the acrylonitrile metabolite, *N*-acetyl-S-(2-cyanoethyl)-l-cysteine (CYMA), and other VOC metabolites, compared with never users, and that daily cigar users had levels comparable to daily cigarette users (Chang et al., n.d.). PATH Waves 1–5 further demonstrated that while cotinine, NNAL and CYMA among exclusive premium cigar users was low due to infrequent use, daily premium cigar users had levels similar to non-premium cigar and cigarette users (Dai et al., 2023). However, prior work has generally grouped cigar users broadly or categorized them only as daily versus non-daily, without accounting for recency of use before biospecimen collection.

This study provides contemporary evidence on biomarkers of exposure among individuals who exclusively smoke cigars, stratified by recency of use. Utilizing PATH Wave 7 (2022–2023), we quantified nicotine and select toxic metal biomarkers (cadmium, lead, uranium), relevant to cardiovascular risk and systemic toxicant exposure, among recent adult exclusive cigar users, stratified by recency of use (smoked on the biospecimen collection day versus 1–2 days earlier), compared to exclusive cigarette and never tobacco users.

## 2 Methods

### 2.1 Study design and population

We analyzed adults (≥18 years of age) from PATH Wave 7. The analytic sample included recent exclusive cigar and cigarette users reporting use within three days preceding biospecimen collection, and never tobacco users. Exclusive cigar users reported cigar smoking (cigarillos, filtered cigars and/or traditional cigars; premium cigars were not differentiated) and no other tobacco/nicotine product use. Exclusive cigarette users reported cigarette smoking and no other tobacco/nicotine product use. Never users reported lifetime non-use of tobacco/nicotine products. Users were classified by last use as “Today” (collection day) or “Yesterday/Day Before” (1–2 days before, but not on, the collection day).

### 2.2 Measures

Demographic characteristics included age (25–34, 35–54 and ≥ 55 years), sex (male, female), race/ethnicity (White non-Hispanic, Black non-Hispanic and other), education (≤some college, college graduate or higher), annual household income (<$50,000, $50,000-100,000, >$100,000) and body mass index (BMI) category. Self-reported cardiovascular conditions and risk factors were derived from survey items asking whether a doctor, nurse, or other health professional ever told participants they had hypertension, hypercholesterolemia, congestive heart failure, stroke, myocardial infarction, atrial fibrillation, or some other heart condition. Tobacco-use variables included cigar subtype, quantity smoked, frequency of days smoked, and use of flavored products.

The CDC Division of Laboratory Sciences conduced assays using validated methods described in the PATH Study Wave 7 Biomarker Restricted-Use Files/codebooks (Metals, DS7073; Urinary Nicotine Metabolites, DS7076) (United States Department of Health and Human Services, 2026). Limits of detection (LOD) were 0.055 μg/L for cadmium, 0.022 μg/L for lead, 0.0024 μg/L for uranium, and 0.03 ng/mL for both cotinine and trans-3′-hydroxycotinine; total nicotine equivalents (TNE-2) was the molar sum of total (free and glucuronide-conjugated) cotinine and trans-3′-hydroxycotinine. Values below LOD were imputed as LOD/√2 (United States Department of Health and Human Services, 2026). Survey-weighted geometric mean concentrations were calculated by group.

### 2.3 Statistical analysis

Survey-weighted linear regression compared log-transformed biomarker levels between groups in unadjusted models and models adjusted for potential confounders such as age, sex, race/ethnicity, income, and BMI (Chang et al., n.d.; Dai et al., 2023). Because metals may contribute to cardiovascular disease (CVD), we conducted sensitivity analyses that additionally adjusted for self-reported CV conditions/risk factors. As a sensitivity analysis, biomarker comparisons were repeated using a 30-day exclusivity definition, restricting to those reporting cigar or cigarette use within 3 days of biospecimen collection and no other tobacco/nicotine product use during the preceding 30 days. Nicotine biomarkers-metal-correlations were estimated from survey-weighted linear regression models. Statistical significance was determined at *p* < 0.05. Analyses accounted for the PATH complex sampling design (balanced repeated replication with Fay's adjustment) to ensure correct variance estimation. Analysis of deidentified data was exempted by UCLA Institutional Review Board.

## 3 Results

The analytic sample included 79 exclusive cigar users, 1192 exclusive cigarette users and 691 never tobacco users (Table 1). Compared with cigarette users, cigar users were predominately male (85.3% vs. 47.3%, *p* < 0.05) and older (61.9% aged ≥55 years vs. 46.3%, *p* < 0.05), and had greater representation of non-Hispanic Blacks (27.1% vs. 21.2%, *p* < 0.05). Cigar users had the highest prevalence of self-reported cardiovascular conditions and risk factors (58.3%), driven primarily by hypercholesterolemia and hypertension, compared with cigarette users (37.3%) and never tobacco users (32.5%; all *p* < 0.05).

**Table 1:** Demographic Characteristics of U.S. Adults by Tobacco Use Status, PATH Study Wave 7 (2022–2023).

*Variables*	Cigar Users *n* = 79	Cigarette Users *n* = 1192	Never Tobacco Users *n* = 691
*Age group*^*a1*^			
25–34	16.53 (6.44, 26.61)	11.22 (9.18, 13.25)	14.75 (11.68, 17.82)
35–54	21.54 (7.96, 35.13)	42.53 (37.87, 47.18)	38.46 (34.11, 42.81)
55+	61.93 (42.29, 81.57)	46.26 (41.57, 50.94)	46.79 (42.06, 51.53)
			
*Sex*^*a2, c2*^			
Male	85.34 (75.04, 95.63)	47.31 (42.53, 52.09)	39.44 (33.97, 44.90)
Female	14.66 (4.37, 24.96)	49.35 (35.06, 63.64)	60.56 (55.10, 66.03)
			
*Race/Ethnicity*^*b2*^			
White, non-Hispanic	44.92 (23.11, 66.73)	61.51 (56.53, 66.49)	61.70 (56.37, 67.04)
Black, non-Hispanic	27.11 (10.67, 43.55)	21.22 (16.69, 25.75)	10.87 (7.79, 13.96)
Other	27.97 (0.00, 60.29)	17.27 (14.04, 20.51)	27.43 (22.57, 32.28)
			
*Education*^*a2,b2*^			
Less than HS/Some College	68.74 (51.15, 86.33)	87.57 (83.66, 91.47)	53.72 (47.44, 60.00)
4+ Years of College	31.26 (13.67, 48.85)	12.43 (8.53, 16.34)	46.28 (40.00, 52.56)
			
*Household Income*^*a1, b2*^			
<$50,000	52.89 (29.64, 76.14)	66.22 (61.54, 70.90)	37.86 (31.32, 44.40)
$50,000-100,000	18.30 (5.44, 31.17)	23.18 (19.80, 26.56)	27.18 (21.73, 32.63)
>$100,000	28.81 (13.56, 44.46)	10.60 (6.96, 14.24)	34.96 (27.90, 42.03)
			
*CV Conditions/Risk Factors*^*a1, c2*^			
No	41.75 (21.12, 62.24)	62.69 (58.52, 66.87)	67.47 (60.90, 74.03)
Yes	58.25 (37.76, 78.74)	37.31 (33.13, 41.48)	32.53 (25.97, 39.10)
			
*BMI*			
Underweight/healthy (<18.5–24.9)	27.81 (11.53, 44.10)	34.70 (29.72, 39.69)	30.16 (24.02, 36.30)
Overweight (25–29.99)	20.57 (8.99, 32.15)	28.30 (23.69, 32.89)	35.53 (28.91, 42.16)
Obese (≥30)	51.61 (28.36, 74.85)	37.00 (32.41, 41.59)	34.31 (29.06, 39.57)

Data are shown as unweighted n and weighted % (95% CI). ^a^cigar vs cigarette; ^b^cigarette vs never; ^c^cigar vs never. Superscript numbers indicate significance level: ^1^*p* < 0.05; ^2^*p* < 0.01; ^3^*p* < 0.001. BMI = body mass index; CV = cardiovascular.

Cigarillos were the most common subtype (42%), followed by filtered (30%) and traditional cigars (28%); some users reported multiple subtypes. Use frequency was generally low, with 72.7% of cigarillo users reporting only one cigarillo in the past 3 days, whereas filtered and traditional cigar users more often reported smoking two or more (63.2% and 57.1%, respectively) in the past 3 days. Flavored cigar products were highly prevalent, with 94% of cigarillo users, 85% of filtered cigar users, and 67% of traditional cigar users indicating that their last cigar smoked was flavored.

Among “Today” cigar users, cadmium was 0.30 μg/L vs 0.16 μg/L (*p* = 0.001), uranium 0.010 μg/L vs 0.005 μg/L (*p* = 0.002), and cotinine and TNE-2 were higher than in never users (*p* < 0.01; Table 2). Compared with never-users, lead was elevated among “Yesterday/Day-Before” cigar users (0.30 μg/L vs 0.24 μg/L; *p* = 0.04), and TNE-2 remained higher (3.11 nmol/mL vs 0.002 nmol/mL; *p* < 0.01).

**Table 2:** Urinary Biomarker Geometric Mean Concentrations Among U.S. Adults by Tobacco Use Group and Recency of Use, PATH Study Wave 7 (2022–2023).

*Biomarkers*	Exclusive Cigar Users		Exclusive Cigarette Users		Never Tobacco Users *n* = 691
	Today *n* = 44	Yesterday/Day Before Yesterday *n* = 35	Today *n* = 1070	Yesterday/Day Before Yesterday *n* = 122	
*Cadmium, μg/L*	0.30 (0.21, 0.44)^cd^	0.12 (0.09, 0.17)	0.35 (0.32, 0.39)^ab^	0.27 (0.19, 0.40)^ef^	0.16 (0.15, 0.18)
*Lead, μg/L*	0.33 (0.23, 0.45)	0.30 (0.23, 0.38)^d^	0.35 (0.33, 0.37)^a^	0.37 (0.26, 0.54)	0.24 (0.21, 0.27)
*Uranium, μg/L*	0.01 (0.007, 0.011)^cd^	0.004 (0.003, 0.006)	0.008 (0.007, 0.009)^abe^	0.01 (0.009, 0.015)^e^	0.005 (0.005, 0.006)
*Cotinine,* μg/L	926.46 (406.47, 2111.66)^cd^	201.38 (86.55, 468.58)^cd^	2812.74 (2544.82, 3108.86)^ab^	744.22 (484.95, 1142.10)^ef^	0.14 (0.11, 0.17)
*Total Nicotine Equivalents-2, nmol/mL*	14.89 (7.34, 30.20)^cd^	3.11 (1.39, 6.96)^cd^	44.56 (40.15, 49.45)^abef^	11.82 (6.92, 20.19)^ab^	0.002 (0.0019, 0.0028)

Data are shown as unweighted n and survey-weighted geometric mean (95% CI). Superscripts indicate pairwise comparisons (*p* < 0.05). ^a,b^cigarette vs never; ^c,d^cigar vs never; ^e,f^cigarette vs cigar; first letter = model without covarites, second = model with covariates. Adjusted models included age, sex, race/ethnicity, household income, and BMI.

Compared with cigarette users on the same timeline, “Today” cigar users had similar cadmium (0.30 vs 0.35 μg/L) and lead (0.33 vs 0.35 μg/L), slightly higher uranium (0.010 vs 0.008 μg/L), and lower TNE-2 (14.89 vs 44.56 nmol/mL). Among those who last used 1–2 days earlier, cigar users had broadly similar cadmium and lead, but lower uranium (0.004 vs 0.012 μg/L) and lower TNE-2 (3.11 vs 11.82 nmol/mL) than cigarette users. In covariate-adjusted models, “Today” cigar users had significantly higher cadmium, uranium, and TNE-2 than never users, whereas “Yesterday/Day-Before” cigar users had higher lead and nicotine biomarkers. Findings from the 30-day sensitivity analysis were broadly similar to the primary analysis, particularly among same-day cigar users (Supplementary Table S1), and additionally adjusting for CV conditions/risk factors, estimated differences in urinary metal concentrations by tobacco use group were not statistically significant (data not shown).

Among exclusive cigarette users reporting use today and yesterday/day before, TNE-2 correlated positively with urinary cadmium (*r* = 0.35, *p* < 0.01; *r* = 0.25, *p* < 0.01, respectively), lead (*r* = 0.19, *p* < 0.01; *r* = 0.20, *p* < 0.01, respectively), and uranium (*r* = 0.08, *p* < 0.01; *r* = 0.01, *p* > 0.05, respectively). In exclusive cigar users today and yesterday/day-before, correlations between TNE-2 and these metals were small and not statistically significant. Correlations were near zero among never-users (*r* ≤ 0.01).

## 4 Discussion

Using nationally representative PATH Wave 7 data, recent exclusive cigar smoking was associated with higher urinary TNE-2 and higher cadmium, lead, and uranium concentrations than never tobacco-users. Among same-day cigar users, cadmium and lead were similar to concentrations observed in exclusive cigarette users, whereas uranium was slightly but significantly higher, and TNE-2 was lower.

These findings extend earlier PATH analyses by showing that biomarker concentrations among exclusive cigar users vary by recency of use. Prior Wave 1 PATH analysis demonstrated that cigar-only users had elevated TNE concentrations, compared to non-tobacco users, although generally lower than daily cigarette users (Chang et al., n.d.). Although TNE-2 levels among cigar users were lower than in same-day and 1–2 day cigarette users, even with generally infrequent use, cigar users demonstrated systemic nicotine uptake, underscoring the importance of sampling timing in product comparisons.

Higher urinary metal levels have been prospectively associated with greater incident CVD and all-cause mortality in the Multi-Ethnic Study of Atherosclerosis (MESA) (Martinez-Morata et al., 2024). Multi-metal analyses in NHANES similarly demonstrate that mixtures of urinary metals are associated with higher CVD and mortality risk, with cadmium and other metals contributing major roles (Guo et al., 2022). In additional MESA analyses, higher urinary uranium levels have been linked to greater progression of coronary artery calcification, a marker of subclinical atherosclerosis (McGraw et al., 2024). The Strong Heart Study reported that higher uranium concentrations were associated with adverse cardiac geometry and impaired left ventricular function in American Indian adults (Lieberman-Cribbin et al., 2024). Using Wave 1 PATH data, Chang et al. reported elevated cadmium and lead among cigar users, with higher levels in daily versus someday users (Chang et al., n.d.). Uranium was not assessed. Whereas prior PATH cigar biomarker studies characterized exposure according to use frequency (e.g., everyday versus someday use) and cigar subtype, our findings extend this evidence by evaluating uranium and classifying very recent cigar use by timing relative to biospecimen collection in PATH Wave 7.

Previous studies have reported biomarker of exposure and oxidative stress differences across cigar sub-types, showing premium cigar users had lower exposures than cigarillo or filtered cigar users (Dai et al., 2023). Our findings align with this pattern: given the high prevalence of flavored use among cigarillo and filtered cigar participants, the relatively high exposures we observe are plausibly consistent with the more intense inhalation/use patterns in that subgroup. A 2022 review of cigar and cigar-type products reported that cadmium and lead are present in cigar tobacco at considerable levels, varying widely by origin and product type (Hać et al., 2022). Our study provides in vivo biomarker evidence that metal exposure from cigar smoking is measurable, and may rival cigarette-based exposure profiles. These data support considering metal exposure in cigar risk assessment.

Longitudinal PATH analyses show that exclusive cigar use, particularly flavored cigar use, is relatively transient and less stable than cigarette use (Buszkiewicz et al., 2024). Despite this instability, our biomarker data show that recent exclusive cigar smoking produces elevations in cadmium, lead and uranium approaching cigarette levels when assessed soon after use, though residual contribution from prior or former cigarette smoking cannot be excluded. Cross-cohort analyses further demonstrated that current and exclusive cigar use was associated with significantly higher levels of systemic inflammatory and prothrombotic markers, including high-sensitivity C-reactive protein, interleukin-6, and fibrinogen, compared with non-tobacco users (Yao et al., 2025). These elevations paralleled those observed among cigarette users, underscoring that cigar use, even when exclusive, appear to contribute to cardiovascular injury. Together, these lines of evidence challenge the perception of cigars as a less harmful alternative to cigarettes (Vargees et al., 2023; Sterling et al., 2016), and support stronger product standards for flavored/non-premium cigars, clear risk communication, and inclusion of cigars in cessation strategies.

This cross-sectional, single wave analysis cannot establish causality or longitudinal biomarker trajectories. Although we stratified users by recency of use, interpretation of biomarker differences should consider the biological half-lives of metals in urine. Cadmium has a relatively long half-life, and lead may be influenced by long-lived skeletal stores; so urinary concentrations reflect cumulative exposure (Suwazono et al., 2009; Rabinowitz et al., 1976), whereas most uranium is eliminated in the urine over 1–2 days after exposure, so levels reflect recent exposure (Brugge et al., 2005). Past-12-month cigarette smoking was reported by 23.5% of cigar users, providing context for potential residual cigarette exposure. Patterns were broadly similar in the 30-day sensitivity analysis; however, more remote cigarette smoking may still contribute, particularly to cadmium and lead, supporting future inclusion of shorter-lived exposure biomarkers. We were unable to disaggregate by cigar subtype or inhalation pattern—factors highlighted as important determinants of systemic toxicant exposure and CVD risk across tobacco products— (Benowitz, 2025), and residual contribution from prior or former cigarette smoking or non-tobacco environmental sources cannot be excluded. Finally, the weak TNE-2-metal correlations may reflect the smaller cigar sample, heterogeneous use, and the longer biological persistence of cadmium and lead relative to TNE-2; these exploratory correlations warrant caution.

## 5 Conclusions

Compared with never tobacco users, recent exclusive cigar smoking is associated with systemic exposure to nicotine and heavy metals at magnitudes approaching those found among cigarette users. While these elevated metal biomarkers raise concern, the actual health risks associated with the specific exposure levels observed here require additional research. While epidemiologic studies have identified cadmium, lead and uranium as associated with CVD incidence and mortality, the extent to which metals explain the overall risks of inhaled tobacco products is unknown. These contemporary biomarker findings challenge perceptions of cigars as less harmful than cigarettes (Vargees et al., 2023; Sterling et al., 2016), and support cigars regulation and cessation efforts.

## Studies in human

This study was performed in compliance with relevant laws, regulatory frameworks and guidelines where the research took place.

The UCLA Institutional Review Board granted an exemption. The authors provided the following explanation: Analysis of deidentified data was exempted by UCLA Institutional Review Board.

## CRediT authorship contribution statement

**Mary Rezk-Hanna:** Writing – original draft, Visualization, Validation, Supervision, Project administration, Methodology, Investigation, Formal analysis, Conceptualization. **Amanda Adolfo:** Writing – review & editing, Writing – original draft, Investigation, Formal analysis, Conceptualization. **Umme S. Warda:** Writing – review & editing, Software, Investigation, Formal analysis, Data curation. **Mary-Lynn Brecht:** Writing – review & editing, Software, Resources, Methodology, Formal analysis, Data curation, Conceptualization. **Neal L. Benowitz:** Writing – review & editing, Validation, Supervision, Methodology, Investigation, Conceptualization.

## Informed consent and patient details

Population Assessment of Tobacco and Health Study: https://pathstudy.nih.gov/participants

Written informed consent to take part in the study and to publish the article has been obtained from all participants or their legal representatives. The privacy rights of participants have been observed.

## Funding

This work was not supported by any external funding.

## Declaration of competing interest

The authors declare the following financial interests/personal relationships which may be considered as potential competing interests: Dr. Benowitz consults with pharmaceutical companies that market or are developing smoking cessation medications and has been a paid expert witness in litigation against tobacco companies. All other authors disclose no conflicts of interest.

## Appendix A Supplementary data

Supplementary material Supplemental Table S1. Thirty-Day Sensitivity Analysis of Uninary Biomarker Geometric Mean Concentrations Among U.S. Adults by Tobacco-Use Group, PATH Study Wave 7 (2022-2023).
