# Supplementary material for: Nicotine and heavy metal exposure in recent exclusive cigar users: Insights from the U.S. population assessment of tobacco and health study wave 7 (2022−2023)
Source: Prev Med Rep. 2026 Oct 3;71:103648. doi: 10.1016/j.pmedr.2026.103648 (PMC13673095; doi:10.1016/j.pmedr.2026.103648)
Supplement: Supplementary file 1 [file mmc1.docx]

**Supplemental Table S1.** Thirty-Day Sensitivity Analysis of Uninary Biomarker Geometric Mean Concentrations Among U.S. Adults by Tobacco-Use Group, PATH Study Wave 7 (2022-2023)

| *Biomarkers* | Exclusive Cigar Users | | Exclusive Cigarette Users | | Never Tobacco  Users  *n* =697 |
| --- | --- | --- | --- | --- | --- |
|  | Today  *n*=46 | Yesterday/Day Before Yesterday  *n* =35 | Today  *n*=1252 | Yesterday/Day Before Yesterday  *n* =126 |  |
| *Cadmium, μg/L*  *Lead, μg/L*  *Uranium, μg/L*  *Cotinine,* µg/L  *Total Nicotine Equivalents-2, nmol/mL* | 0.36 (0.27, 0.48)^cd^  0.34 (0.23, 0.52)^cd^  0.01 (0.008~~4~~, 0.01)^cd^  971.92 (457.26, 2065.85)^cd^  16.76 (8.98, 31.27) | 0.12 (0.08, 0.19)^cd^  0.28 (0.19, 0.41)^cd^  0.004 (0.003, 0.006)^cd^  337.33 (175.38, 648.82)^cd^  4.89 (2.55,9.39)^cd^ | 0.37 (0.34, 0.40)^ab^  0.36 (0.33, 0.38)  0.008 (0.007, 0.009)^abe^  2955.05 (2714.19, 3217.30)^abef^  45.73 (42.28, 49.46)^abef^ | 0.25 (0.17, 0.38)^e^  0.37 (0.26, 0.53)  0.009 (0.006, 0.013)^ef^  630.85 (290.25, 1371.14)^ab^  10.29 (4.71, 22.51)^ab^ | 0.16 (0.15, 0.18)  0.24 (0.21, 0.27)  0.005 (0.005, 0.006)    0.14 (0.11, 0.17)  0.002 (0.002, 0.003) |

Data are shown as unweighted n and survey-weighted geometric mean (95% CI). Superscripts indicate pairwise comparisons (*p*<0.05). ^a,b^cigarette vs never; ^c,d^cigar vs never; ^e,f^cigarette vs cigar; first letter = model without covarites, second = model with covariates. Adjusted models included age, sex, race/ethnicity, household income, and BMI. Groups were defined using past-30-day tobacco-use criteria; Ns therefore differ from the primary analysis.
